# Predictors of increased acute postoperative pain after elective minimally invasive colorectal surgery

**DOI:** 10.1007/s00384-026-05119-5

**Published:** 2026-03-10

**Authors:** Mario Kaufmann, Vanessa Orth, Tim-Janick Dorwarth, Florian Herrle, Christoph Reißfelder, Julia Hardt

**Affiliations:** Department of Surgery, Medical Faculty Mannheim, University Medical Centre Mannheim, Heidelberg University, Theodor-Kutzer-Ufer 1-3, 68167 Mannheim, Germany

**Keywords:** Acute postoperative pain, Colorectal surgery, Transversus abdominis plane block, Enhanced recovery after surgery

## Abstract

**Purpose:**

Postoperative acute pain is a major obstacle to archiving key goals in modern perioperative treatment concepts such as ERAS® (enhanced recovery after surgery). Despite a multimodal pain management concept, some patients continue to suffer from severe pain. The aim of this analysis is to identify predictors of severe postoperative pain following elective minimally invasive intestinal surgery.

**Methods:**

Data from 49 patients, who underwent intestinal resection between April 2021 and March 2022 were used for this purpose. Various pre- and intraoperative characteristics were examined for their influence on pain in the morning in a univariate and multivariate analysis. Increased postoperative pain is defined by a NRS (numerical rating scale) of at least 4 at rest.

**Results:**

It was found that patients with severe postoperative pain (*n* = 16) on the first postoperative day (POD) had a significantly higher BDI (Beck Depression Index) score of 16.1 (± 10.46) compared to patients without severe postoperative pain (*n* = 33) with 8.89 (± 7.03) (*p* = 0.007). In the multivariate analysis, the BDI score was also significant with an Odds Ratio of 1.14 (CI 95% 1.02-1.29, *p* = 0.002). On POD 2, patients with increased pain (*n* = 10) were significantly younger (53.1 years (± 16.40)) than patients without increased pain (*n* = 39) (65.8 years (± 12.64)) (*p* = 0.01). This was also confirmed in the multivariate analysis with an Odds Ratio of 1.12 (CI 95% 1.02-1.24, *p* = 0.019).

**Conclusion:**

It was demonstrated that a younger age, higher BDI score and the presence of IBD are significant predictors of severe postoperative pain despite multimodal pain management.

**Supplementary Information:**

The online version contains supplementary material available at 10.1007/s00384-026-05119-5.

## Introduction

Multimodal pain therapy has been firmly established in colorectal surgery for many years and is recommended in the current ERAS® (enhanced recovery after surgery) recommendations from 2025 and the German POMGAT (Perioperative Management of Gastrointestinal Tumors) guideline [7, 19]. Adequate pain management can accelerate postoperative recovery in terms of faster mobilization. restoration of gastrointestinal functions and early dietary rehabilitation, and reduce complications (e.g. pneumonia, thrombosis) [7]. This leads to a shorter hospital stay (LoS).

The transverse abdominis plane block (TAPB) compared to the thoracic epidural anaesthesia (TEA) gives an advantage of a significant reduction in the rate of hypotension, PONV (Postoperative Nausea and Vomiting), ileus and paraesthesia [2, 18, 25] and results in a shortened LoS [1, 2, 4, 25]. As a result, TAPB is the procedure of choice in the current ERAS® guideline from 2025, and TEA is no longer recommended in minimally invasive colorectal surgery [7]. Both ultrasound-guided TAPB (US-TAPB) and laparoscopic TAPB (L-TAPB) are recommended, as both procedures have proven to be equally effective in colorectal surgery [7, 9]. However, one advantage of L-TAPB is that it can be applied more quickly [5].

In a previous study, we were able to show that a two-stage laparoscopic visually controlled TAPB (TS-L-TAPB) is an effective procedure for reducing the requirement for morphine equivalents [10]. We realized that a strictly correct application led to a significant reduction in pain already in the recovery room [11].

We observed in the study, that many patients experienced just minor pain in the recovery room and on the first day after surgery. The mean and median values were below during this period in the hospital's internal threshold (≥ 4) for administering opiates when L-TAPB was performed correctly in two stages.

However, there were also some patients in our study cohort who complained of increased pain despite adequate performance of TS-L-TAPB and adherence to the standard pain management regimen. In a meta-analysis, Yang et al. were able to establish that younger patients, women, smokers, depression, anxiety, sleep disorders, a higher BMI, existing preoperative pain and existing preoperative analgesia were risk factors for inadequate postoperative pain management after any surgical procedure [27]. However, there are no comparable studies that explicitly examine elective colorectal surgery.

For gastrointestinal procedures involving both the upper and lower gastrointestinal tract, independent risk factors for increased postoperative pain include oral opiate use, preoperative anxiety and anticipated postoperative pain [14]. It has been reported that younger patients, in particular, experience more severe postoperative pain following colorectal surgery. Also in acute postoperative pain after haemorrhoidectomy, younger age, especially <40 years, increased preoperative pain, the use of a tamponade, the absence of a pudendal block and a longer operation time were identified as predictors of increased postoperative pain [15]. Increased and persistent acute postoperative pain is a risk factor for the development of chronic pain [6]. The incidence of chronic pain after colorectal surgery is reported to be 17–21% [22].

The aim of this analysis is to detect predictors of increased postoperative pain (NRS score ≥ 4) after minimally-invasive colorectal surgery despite TS-L-TAPB.

## Methods

Between April 2021 and March 2022, all adult patients who underwent minimally invasive intestinal surgery at Mannheim University Hospital were consecutively included. A new L-TAPB technique was implemented and tested in this study cohort. This allowed us to monitor all patients closely. In accordance with the exclusion criteria of the original studies, patients with a GFR <30, ASA >3, a language barrier, non-compliant patients, patients undergoing morphine or cannabis therapy (as an indication of chronic severe pain), or psychological stress were excluded. For the latter, the DASS (Depression Anxiety Stress Scales, threshold values depression ≥ 10, anxiety ≥ 6, stress ≥ 10) and the BDI-V (Beck-Depressions-Inventar)for depression (threshold value > 35 points) were performed [17, 21]. BDI values ≤ 35 were analysed as part of the survey. Patients were also excluded if a TEA was inserted preoperatively or if a conversion to open surgery was performed. Additionally, only patients for whom L-TAPB was correctly performed according to the hospital standard were analysed. In May 2021, further training was offered to all surgeons at the clinic. In 49 patients a correct L-TAPB was performed as described previously [10, 11].

Intraoperatively, all patients received the same in-house anesthesiologic standard for colorectal surgery. The regimen consisted of thiopental 3–5 mg/kg (alternatively propofol 2 mg/kg) and sufentanil 0.3–0.5 µg/kg. In addition, patients received 2 mg metamizole and 0.5 mg/kg S-ketamine as a short infusion 15 minutes before suturing.

In the recovery room, patients received 1000 mg of paracetamol or metamizole, 5 mg of oxycodone or 7.5 mg of piritramide, depending on the level of pain.

The in-house standard on the ward was standard medication consisting of oral intake of 1000 mg of metamizole four times a day. If necessary, patients could receive an additional 1000 mg of paracetamol, 5–10 mg of oxycodone or 7.5 mg of piritramide. The specified goal for postoperative pain was to achieve pain control with an NRS ≤ 3. Pain was routinely assessed three times a day by the nurses and more frequently if necessary.

All patients were treated according to the same standardized ERAS® pathway, which was based on the ERAS® guideline from 2018 that was valid at the time.

Jamovi (R-Core) was used for statistical analysis. Categorical data are presented as absolute and relative frequencies and were analysed using the chi-square or Fisher's exact test. For continuous data, means and standard deviations or medians and minimum/maximum values are calculated, depending on the distribution. The comparison was performed using the t-test or Mann-Whitney U-test, depending on the distribution. Multivariate logistic regression was performed to estimate possible associations between predictors and the NRS. The results are presented as Odds Ratios (OR) with 95% confidence intervals.

The original study was prospectively registered in the German Clinical Trials Register (DRKS number DRKS00024839) and approved by the Ethics Committee II of the University of Heidelberg (No. 2021-503).

## Results

**Fig. 1:**
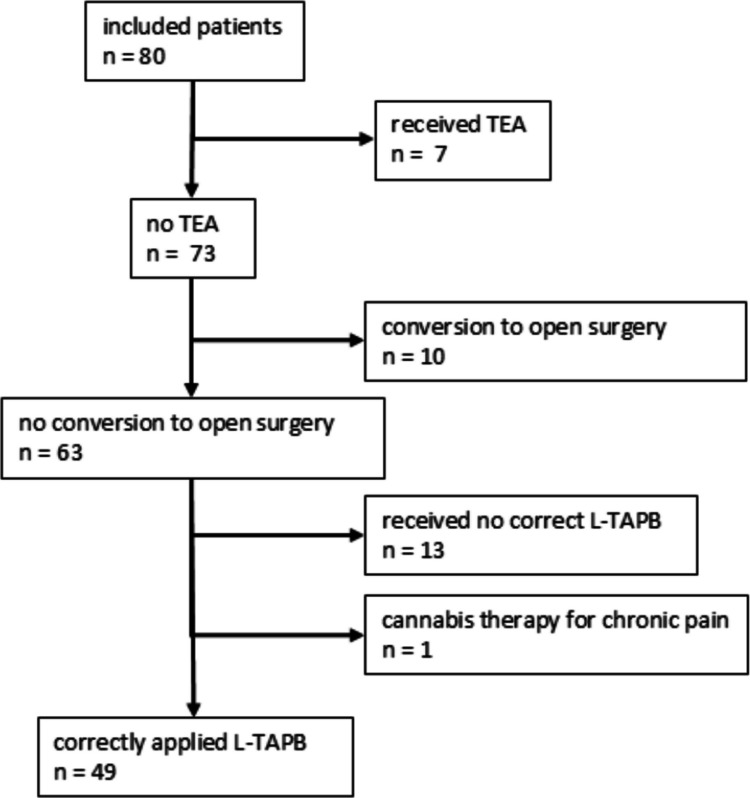
Patients included in the initial prospective arm of the study, which could now be used for this subgroup analysis

A total of 133 patients were screened as part of the study. 89 patients met the inclusion criteria for the study, and ultimately 80 patients were included in the study. Of these patients, 10 underwent conversion to open surgery, 7 received a TEA and 13 did not receive an L-TAPB (Fig 1). A detailed description of the included and excluded patients can be found in the previously published paper on the effectiveness of L-TAPB compared to TEA [10]. During data collection, it emerged that one patient underwent a cannabis therapy for chronic pain. Ultimately, 49 patients could be analysed for the study question. The basic data can be seen in Table 1.

**Table 1:** Basic data on the patient population

Age (mean, SD) (median, min.-max) (years)		63.2	(± 14.27)	65	(28–88)
Sex (n, %)					
	men	25		51%	
	women	24		49%	
BMI (mean, SD) (median, min.-max) (kg/m^2^)		26,4	(± 4.88)	25	(18–40)
ASA class (n, %)					
	ASA I	9		18.4 %	
	ASA II	36		73.5 %	
	ASA III	4		8.2 %	
Location of procedure (n, %)					
	Small bowel/colon	15		30.6 %	
	rectum	34		69.4 %	
Indication (n, %)					
	neoplasia	38		77.6 %	
	IBD	4		8.2 %	
	diverticulitis	7		14.3 %	
Minimal-invasive technique (n, %)					
	laparoscopic	44		89.8 %	
	robotic	5		10.2 %	
BDI (mean, SD) (median, min.-max.)		11	(± 8.7)	10	(0–32)
Operating time (mean, SD) (median, min.-max) (minutes)		245,4	(± 114.67)	222	(96–568)
Stoma creation (n, %)		8		16.3%	

*SD* standard deviation, *min*. minimum, *max*. maximum, *BMI* body mass index, *ASA* American Society of Anesthesiologists, *IBD* inflammatory bowel disease, *BDI* Becks-Depression-Index

### Recovery room

In the recovery room, the NRS averaged 2.12 (± 2.31) and the median was 1 (0–7) (Table 2). Severe pain ≥ 4 was reported in 32.7% (*n* = 16) of patients (Table 3). The univariate analysis showed that the surgical indication, especially IBD, had a significant influence on increased postoperative pain (*p* = 0.035) (Table 4). In the multivariate analysis, no predictor was found to be significant.

**Table 2:** Average and median pain scores from the numerical rating scale

	Mean (standard deviation)		Median (minimum-maximum)	
Recovery room	2.12	(± 2.31)	1	(0–7)
POD 1, 8 a.m.	2.88	(± 2.03)	3	(0–8)
POD 2, 8 a.m.	2.02	(± 1.85)	2	(0–8)
POD 3, 8 a.m.	1.12	(± 1.35)	0	(0–5)

*POD* postoperative day

**Table 3:** Percentage of patients with mild (NRS 0–3) and severe pain (NRS 4–10)

	NRS 0–3		NRS 4–10	
Recovery room	33	67.3 %	16	32.7 %
POD 1, 8 a.m.	35	71.4 %	14	28.6 %
POD 2, 8 a.m.	39	79.6 %	10	20.4%
POD 3, 8 a.m.	46	93.9 %	3	6.1 %

*POD* postoperative day, *NRS* Numerical Rating Scale

**Table 4:** Univariate analysis of the influence of baseline data on pain in the recovery room

		NRS 0–3 (*n* = 33)		NRS 4–10 (*n* = 16)		*p*-value
Age (mean, SD) (years)		64	(± 13.68)	61,6	(± 15.75)	0.576*
Sex (n, %)						
	men	17	52%	8	50%	1**
	women	16	48%	8	50%	
BMI (median, min. - max.) (kg/m^2^)		26	(18–40)	24.5	(20–33)	0.285***
ASA class (n, %)						
	I	4	12%	5	31%	0.267****
	II	26	79%	10	62%	
	III	3	9%	1	6%	
Location of procedure (n, %)						
	Small bowel/colon	23	70%	11	69%	1**
	rectum	10	30%	5	31%	
Indication (n, %)						
	neoplasia	25	76%	13	81%	0.035****
	IBD	1	3%	3	19%	
	diverticulitis	7	21%	0	0%	
Minimal-invasive technique (n, %)						
	laparoscopic	30	91%	14	88%	1**
	robotic	3	9%	2	12%	
BDI (median, min. - max.)		9	(0–32)	12	(0–31)	0.381***
Operating time (median, min. - max.) (minutes)		199	(96–568)	292	(120–536)	0.086***
Stoma creation (n, %)						
	Yes	4	12%	4	25%	0.411**
	No	29	88%	12	75%	

*NRS* numerical rating scale, *SD* standard deviation, *min*. minimum, *max*. maximum, *BMI* body mass index, *ASA* American Society of Anesthesiologists, *IBD* inflammatory bowel disease, *BDI* Beck Depression Index, significant *p*-value highlighted, * t-test, ** Fisher's exact test, *** u-test, **** chi-square test

### POD 1 8 a.m.

On the first postoperative day, patients experienced the most severe pain on average, with a score of 2.88 (± 2.03) (Table 2). The median also showed the highest value in the study period at 3 (0–8) (Table 3). This time, the univariate analysis showed that patients with increased postoperative pain had a significantly higher BDI score than 16.1 than patients with low pain (8.89) (*p* = 0.007) (Table 5). The multivariate analysis showed that an elevated BDI score led to higher postoperative pain with an Odds Ratio of 1.14 (CI 95% 1.02–1.29.02.29, *p* = 0.023).

**Table 5:** Univariate analysis of the influence of baseline data on pain on POD 1 8 a.m,

		NRS 0–3 (*n* = 35)		NRS 4–10 (*n* = 14)		*p*-value
age (mean, SD) (years)		65.09	(± 12.08)	58,6	(± 18.38)	0.151*
Sex (n, %)						
	men	18	51%	7	50%	1**
	women	17	49%	7	50%	
BMI (mean, min. - max.) (kg/m^2^)		26	(18–40)	24.5	24.5	0.239***
ASA (n, %)						
	I	7	20%	2	14%	0.873****
	II	25	71%	11	79%	
	III	3	9%	1	7%	
Location of procedure (n, %)						
	Small bowel/colon	25	71%	9	64%	0.735**
	rectum	10	29%	5	36%	
Indication (n, %)						
	neoplasia	29	83%	9	64%	0.097****
	IBD	1	3%	3	21%	
	diverticulitis	5	14%	2	14%	
Minimal-invasive technique (n, %)						
	laparoscopic	32	91%	12	86%	0.616**
	robotic	3	9%	2	14%	
BDI (median, min. - max.)		8,89	(± 7.03)	16,1	(± 10.46)	0.007*
Operating time (median, min. - max.) (minutes)		213	(96–568)	274,5	(131–536)	0.144***
Stoma creation (n, %)						
	Yes	4	11%	4	29%	0.202**
	No	31	89%	10	71%	

*NRS* numerical rating scale, *SD* standard deviation, *Min*. minimum, *Max*. maximum, *BMI* body mass index, *ASA* American Society of Anesthesiologists, *IBD* inflammatory bowel disease, *BDI* Beck Depression Index, significant *p*-value highlighted, * t-test, ** Fisher's exact test, *** u-test, **** chi-square test

### POD 2 8 a.m.

On the second postoperative day, the average pain score was 2.02 (± 1.85), which was lower than the previous day (Table 2). In addition, the median was again 2 (0–8) (Table 3). Patients with increased pain were on average younger (53.1 years) than patients with compensated pain (65.8 years) (*p* = 0.01). In addition, as in the recovery room, the surgical indication was also a significant influencing factor this time (*p* = 0.018) (Table 6). In the multivariate analysis, age was significant this time with an Odds ratio of 1.12 (CI 95% 1.02–1.24.02.24, *p* = 0.019).

**Table 6:** Univariate analysis of the influence of baseline data on pain on POD 2 8 a.m

		NRS 0–3 (*n* = 39)		NRS 4–10 (*n* = 10)		*p*-Wert
Age (mean, SD) (years)		65,8	(± 12.64)	53.1	(± 16,40)	0.01*
Sex (n, %)						
	men (n, %)	19	49%	6	60%	0.725**
	women (n, %)	20	51%	4	40%	
BMI (median, min. - max.) (kg/m^2^)		25	(18–37)	26	(20–40)	0.784***
ASA (n, %)						
	I	7	18%	2	20%	1****
	II	29	74%	7	70%	
	III	3	8%	1	10%	
Location of procedure (n, %)						
	Small bowl/colon	26	67%	8	80%	0.702**
	rectum	13	33%	2	20%	
Indication (n, %)						
	neoplasia	32	82%	6	60%	0.018****
	IBD	1	3%	3	30%	
	diverticulitis	6	15%	1	10%	
Minimal-invasive technique (n, %)						
	laparoscopic	34	87%	10	100%	0.569**
	robotic	5	13%	0	0%	
BDI (median, min. - max.)		10	(0–31)	6	(0–32)	0.535***
Operating time (median, min. - max.) (minutes)		213	(96–465)	251	(131–568)	0.372***
Stoma creation (n, %)						
	Yes	6	15%	2	20%	0.659**
	No	33	85%	8	80%	

*NRS* numerical rating scale, *SD* standard deviation, *Min*. minimum, *Max*. maximum, *BMI* body mass index, *ASA* American Society of Anesthesiologists, *IBD* inflammatory bowel disease, *BDI* Beck Depression Index, significant *p*-value highlighted, * t-test, ** Fisher's exact test, *** u-test, **** chi-square test

**Table 7:** Univariate analysis of the influence of baseline data on pain on POD 3 8 a.m

		NRS 0–3 (*n* = 46)		NRS 4–10 (*n* = 3)		*p* - value
Age (mean, SD) (years)		64,5	(± 12.67)	44,3	(± 26.58)	0.016*
Sex (n, %)						
	men	23	50%	2	67%	1**
	woman	23	50%	1	33%	
BMI (median, min. - max.) (kg/m^2^)		26	(18–40)	22	(21–25)	0.121***
ASA (n, %)						
	I	8	17%	1	33%	0.612****
	II	34	74%	2	67%	
	III	4	9%	0	0%	
Location of procedure (n, %)						
	Small bowl/colon	31	67%	3	100%	0.543**
	rectum	15	33%	0	0%	
Indication (n, %)						
	neoplasia	37	80%	1	33%	0.021****
	IBD	2	4%	2	67%	
	diverticulitis	7	15%	0	0%	
Minimal-invasive technique (n, %)						
	laparoscopic	41	89%	3	100%	1**
	robotic	5	11%	0	0%	
BDI (median, min. - max.)		10	(0–32)	16	(2–25)	0.,587***
Operating time (median, min. - max.) (minutes)		225	(96–568)	133	(131–536)	0.632*
Stoma creation (n, %)						
	Yes	8	17%	0	0%	1**
	No	38	83%	3	100%	

*NRS* numerical rating scale, *SD* standard deviation, *Min*. minimum, *Max*. maximum, *BMI* body mass index, *ASA* American Society of Anesthesiologists, *IBD* inflammatory bowel disease, *BDI* Beck Depression Index, significant *p*-value highlighted, * t-test, ** Fisher's exact test, *** u-test, **** chi-square test

### POD 3 8 a.m.

On the morning of the third postoperative day, the mean NRS was 1.12 (± 1.35). The median was 0 (0–5). Once again, Patients with higher levels of pain were also significantly younger (*p* = 0.016). In addition, it was shown again that the surgical indication had a significant influence on the level of postoperative pain (*p* = 0.021). In the multivariate analysis, none of the variables showed a significant influence Table 7.

## Discussion

Postoperative pain is a key factor influencing patients' postoperative recovery. Pain plays an important role, particularly in the context of the ERAS® concept, which focuses on early mobilisation and nutrition of the patient, as it significantly slows down the patient's recovery of everyday skills.

Multimodal pain therapy is strongly recommended by the current POMGAT and ERAS® guidelines [7, 19]. The L-TAPB has established itself as the central regional anaesthesia procedure for this purpose [7].

The aim of the analysis is to identify patients, whose standard pain therapy was not effective. These patients represent a risk group that requires more extensive or invasive measures. It has already been demonstrated that strictly correct application of L-TAPB leads to a significant improvement in perceived pain [11]. The procedure has proven to be safe and quick to learn, without any major technical difficulties [11]. Incorrect application has been attributed to the surgeon's negligence.

This analysis showed that in the recovery room, the severity of postoperative pain is most significantly influenced by the diagnosis of IBD. This patient group generally suffers from chronic pain at a rate of 30–50% for many years, which is a risk factor for acute postoperative pain [8, 27]. This parameter was generally excluded from the analysis, but the diagnosis nevertheless proved to be a relevant risk factor.

On the first postoperative day, the psychological stress of the patients, measured by the BDI, had a significant influence on postoperative pain. Both the mean value and the median classification as well as the multivariate analysis showed significant values. This is also reflected in the literature [27]. However, patients with a score of ≥ 35 points and thus suffering from depression were excluded [21]. As already described by Yang et al. and Liu et al., various psychological stresses (depression, anxiety and stress) have an influence on postoperative pain [13, 27]. However, the data shows that even the slightest deviations can increase postoperative pain due to psychological stress. Patients with psychological stress, even if only mild, should be identified preoperatively. The questionnaires described here are quick and easy to complete.

As the initial pain from the operation subsided, the univariate analysis showed that a younger age and a diagnosis of IBD were significant factors influencing more severe postoperative pain. On the second postoperative day, the multivariate analysis showed a significant influence of age, which is also described in the literature [15, 27]. It has been shown, that with increasing age, there is a loss of pain sensitivity accompanied by an increase in the pain threshold [12]. In addition, a decrease in kidney function in old age leads to an accumulation of analgesics [23].

Interestingly, the parameters further examined in this study in terms of type of surgery (small bowel, colon, rectum) and additional stoma creation did not lead to increased pain, although rectal surgery, which was mostly combined with a stoma, resulted in a larger wound area. Due to the small number of cases in the study, it was not possible to draw a clear distinction between colon and small bowel resections, so these were analysed in one group.

The surgical procedure (laparoscopic/robotic) also showed no deviations, even though robotic procedures sometimes exert enormous force or stress on the abdominal wall. In addition, the lack of a clinically and statistically relevant difference can also be explained by the small number of robotic surgeries (*n* = 5, 10%).

Due to the significant impact of pain on postoperative recovery, it is important to evaluate which measures can be used to support patients. Patient education plays a crucial role here, which is carried out by ERAS® nurses. This preoperative counselling can reduce anxiety about the operation, which are a potential risk factor for increased postoperative pain [6, 7]. Patients are prepared for pain management even before surgery [16, 26]. The psyche already plays an important role in prehabilitation measures, which are also recommended in the ERAS® guidelines [7]. Preoperative support measures can already have an impact on the significant risk factor of psychological stress.

Another consideration is how perioperative analgesia can be improved. In addition to classic TEA, minimally invasive local anaesthesia procedures play an increasingly important role in modern colorectal surgery. L-TAPB has already found its way into the guidelines, but the best position, timing and medication, have not been found yet.

It is also possible to combine L-TAPB with other procedures. This regional anaesthesia procedure is limited by the maximum doses of local anaesthetics, as these are cardiotoxic [24].

Another option, that has not yet been extensively researched, are anatomical nerve blocks. Here, a positive effect of the celiac plexus block (CPB) has already been demonstrated for bariatric surgery [3]. In colorectal surgery, particularly rectal surgery, the superior hypogastric plexus block (SHPB) may have a positive effect in numbing more visceral pain [20].

## Limitations

The main limitation of the study is the small number of cases, with only 49 patients. However, we decided to carry out this analysis anyway to identify potential high-risk patients who, despite our in-house standards, were still experiencing inadequate pain relief. In addition, patients with chronic pain, which would also have had a high probability of influencing postoperative pain, were excluded.

## Conclusion

This follow-up analysis could define risk factors for acute postoperative pain after colorectal surgery. It showed that younger age, an elevated BDI score indicating depressive stress, and a diagnosis of IBD have a significant influence on the occurrence of increased acute pain. Although the findings of this study are consistent with the current literature, they must be viewed critically due to the small number of cases.

## Supplementary Information

Below is the link to the electronic supplementary material.

Supplementary file1 (DOCX 34 KB)

## Data Availability

The data that support the findings of this study are available on reasonable request from the corresponding author (Julia Hardt).
